# Clinical-radiological dissociation in a patient with nitrous oxide-induced subacute combined degeneration: a case report

**DOI:** 10.1186/s12883-020-01685-5

**Published:** 2020-03-17

**Authors:** Jiwei Jiang, Xiuli Shang

**Affiliations:** Department of Neurology, the First Affiliated Hospital of China Medical University, Number 155, Nanjing Street, Heping District, Shenyang City, 110001 Liaoning Province China

**Keywords:** Subacute combined degeneration, Nitrous oxide, Vitamin B_12_ deficiency, Homocysteine, Magnetic resonance imaging

## Abstract

**Background:**

Several recent studies have reported subacute combined degeneration (SCD) induced by nitrous oxide (N_2_O) abuse. However, the association between the evolution of dynamic neuroimaging and clinical manifestations has not been reported in patients with N_2_O-induced SCD.

**Case presentation:**

We described the case of a 24-year-old man who developed SCD with inverted V-sign hyperintensities over the posterior aspect of the spinal cord caused by frequent, excessive N_2_O inhalation. One month after treatment, his weakness and paresthesia resolved and serum vitamin B_12_ levels exceeded the normal levels. However, the hyperintensities had extended horizontally and longitudinally on T2-weighted magnetic resonance imaging (MRI), compared to those on the initial scan. Two months after treatment, the patient experienced some residual numbness in the distal limbs, and his serum homocysteine levels were normal, but the abnormal signals seen on cervical T2-weighted MRI had decreased only slightly compared to those seen on the one-month follow-up MRI. The evolution of conventional MRI findings lagged compared to the clinical manifestation, which was suggestive of a clinical-radiological dissociation.

**Conclusions:**

Clinical-radiological dissociation might have occurred in this case because T2-weighted imaging was not sensitive enough to reveal cytotoxic edema. Moreover, the serum vitamin B_12_ level is not a good indicator of cellular vitamin B_12_. Thus, clinicians should recognize this phenomenon, comprehensively assess the condition of patients with N_2_O-induced SCD, and avoid terminating treatment based on the resolution of clinical symptoms and serological results.

## Background

Subacute combined degeneration (SCD) is a neurological complication of vitamin B_12_ deficiency, which is typically observed in elderly individuals with malabsorption syndromes and inadequate intake or bioavailability of vitamin B_12._ [1]. Recently, several sporadic cases of otherwise healthy young adults with SCD induced by nitrous oxide (N_2_O) abuse have been described [2]. However, to the best of our knowledge, none of these studies have reported a relationship between the evolution of dynamic neuroimaging and clinical manifestations in a patient with N_2_O-induced SCD. Treatment is frequently discontinued by individuals who abuse N_2_O following improvement in the neurological symptoms [3]. Moreover, guidelines for treatment duration have not been established for patients with SCD. Therefore, we reported the case of a young man diagnosed with SCD caused by extensive N_2_O inhalation that led to clinical-radiological dissociation. Furthermore, we highlighted the fact that the condition of patients with N_2_O-induced SCD cannot be determined by imaging abnormalities, clinical manifestations, or serum vitamin B_12_ levels alone.

## Case presentation

**Fig. 1:**
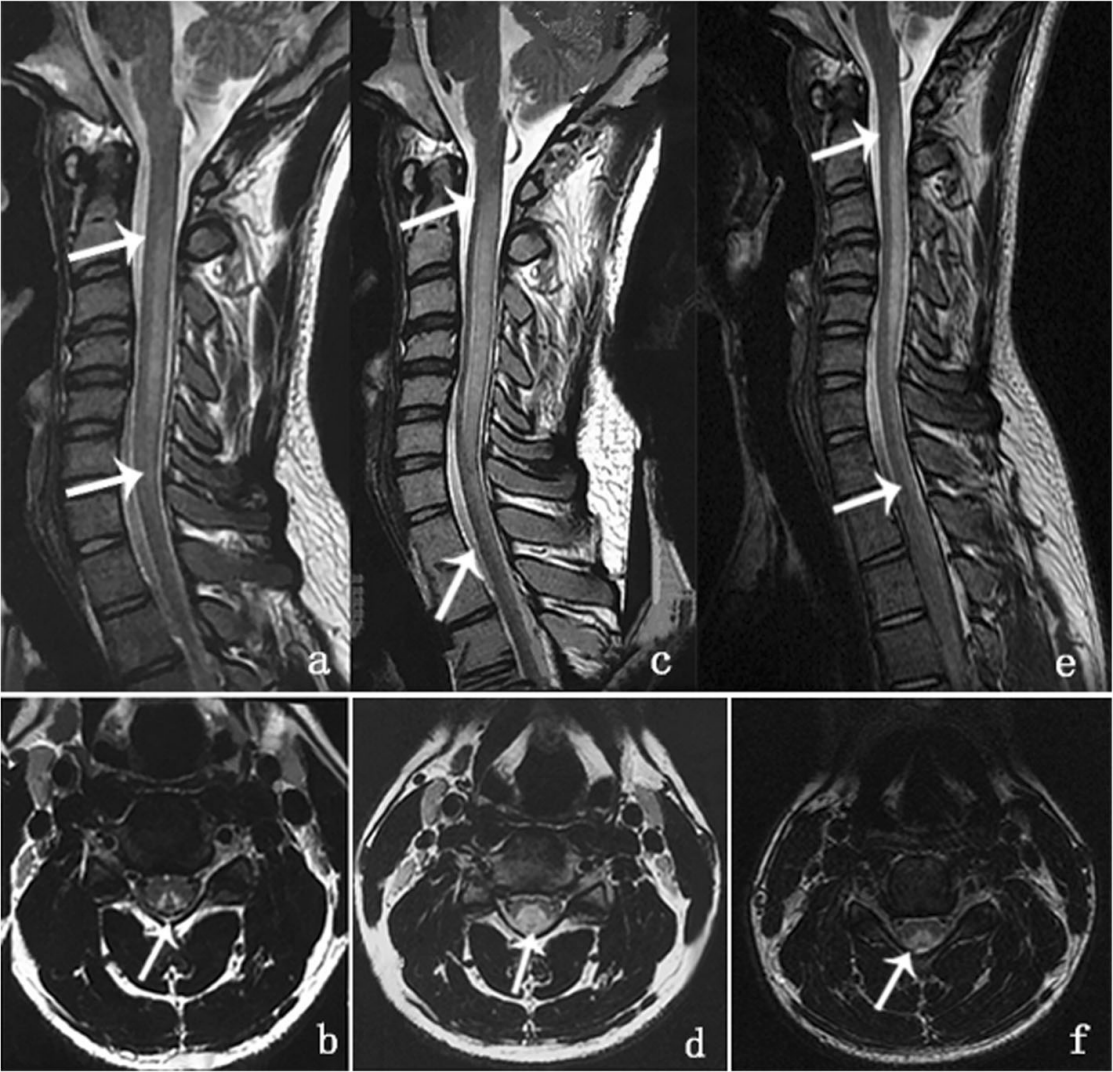
Magnetic resonance imaging of the cervical spinal cord. **a**. Sagittal T2-weighted imaging showed increased intramedullary signal intensity along the posterior column of the spinal cord extending from C2 to C6 on August 3, 2018. **b**. A V-shaped hyperintensity on axial T2-weighted imaging at the C4 level was seen within the dorsal cervical spinal cord on August 3, 2018. **c**. Sagittal T2-weighted imaging showed abnormal, longitudinally and horizontally extensive hyperintensities involving the lateral and posterior columns of the spinal cord extending from C1 through T2 on September 3, 2018. **d**. Axial T2-weighted imaging at the C4 level showed a ball-shaped hyperintensity on September 3, 2018. **e**. Sagittal T2-weighted imaging showed hyperintensity along the posterior column of the spinal cord extending from C1 to T1 on October 4, 2018. **f**. Axial T2-weighted imaging at the C4 level showed that the V-shaped hyperintensity had decreased compared to that in September (**d**)

A 24-year-old man in a wheel-chair presented with numbness in all extremities and worsening lower-extremity weakness for approximately 20 days. He had inhaled N_2_O through an average of approximately 100–200 “whippit” cartridges per day for at least 3 months for recreational purposes. The patient had good dietary intake without alcohol use and did not have a history of smoking or illicit drug use. Neurological examination revealed clear consciousness with fluent speech*.* No abnormalities were detected in the cranial nerves. However, mild weakness in the upper limbs (grade 4), severe weakness in the lower limbs (grade 3), marked increase in the deep tendon reflexes, impaired joint position and vibration sensation, sensory ataxia, positive bilateral Babinski sign, and positive Romberg and Lhermitte’s signs were observed on neurological examination. Laboratory tests revealed decreased levels of serum red blood cell (RBC) (3.32 × 10^12^ /L, reference range: 4.30–5.80 × 10^12^ /L), hemoglobin (Hb) (118.4 g/L, reference range: 130–175 g/L), vitamin B_12_ (98.2 pmol/L, reference range: 145–637 pmol/L), and folic acid (8.38 nmol/L, reference range: 8.83–60.80 nmol/L). Serum homocysteine (Hcy) was considerably elevated (> 50 μmol/L, reference range: 5.46–16.20 μmol/L), which was indicative of functional vitamin B_12_ deficiency at the cellular level. Cerebrospinal fluid (CSF) assessment yielded normal results. The inflammatory, infectious, and immune biomarkers in CSF and serum were unremarkable. Sagittal spinal cord magnetic resonance imaging (MRI) revealed hyperintensities involving the posterior columns from C2 to C6 on T2-weighted images (Fig. 1a), with an inverted V-sign on axial MRI (Fig. 1b). Brain and thoracic MRI findings were normal.

The patient was diagnosed with SCD of the spinal cord induced by N_2_O abuse. Treatment with a high dose of supplementary intramuscular vitamin B_12_ injections (1.5 mg per day), oral folic acid (15 mg per day), and abstinence from N_2_O led to a gradual improvement in the patient’s symptoms. One month later, the symptoms of weakness and paresthesia were resolved; the patient could walk unsupported with some residual gait impairment. The serum RBC, Hb, and folic acid levels improved to normal, and the serum vitamin B_12_ level increased to more than 1476 pmol/L (the maximum measurable value). However, the patient’s serum Hcy remained elevated (19.02 μmol/L). At this time, we observed an interesting phenomenon. The hyperintensities on T2-weighted images had extended, both horizontally and longitudinally, from C1 to T2 (Fig. 1c), resembling a “ball” on the axial images, despite the improvement in the patient’s clinical symptoms and laboratory values (Fig. 1. d). Importantly, the patient had had no exposure to hormones or N_2_O. He was discharged with a prescription for vitamin B_12_ supplements. The patient’s gait had improved, and he experienced only mild paresthesia of the distal limbs during the two-month follow-up. Serum Hcy had improved to normal levels. Moreover, the abnormal signals seen on T2-weighted images had decreased compared to those on the one-month follow-up MRI, but were still more extensive than those seen on the initial MRI (Fig. 1 e, f). It seemed that a clinical-radiological dissociation had occurred, and conventional MRI findings had consistently lagged behind the clinical and laboratory manifestations.

## Discussion and conclusions

Although several cases of SCD associated with vitamin B_12_ deficiency induced by N_2_O abuse have been described, the relationship between the evolution of dynamic neuroimaging and clinical manifestations has never been reported. To the best of our knowledge, this is the first report of clinical-neuroimaging dissociation in a patient with N_2_O-induced SCD.

N_2_O induces SCD by irreversibly oxidizing the cobalt ion of vitamin B_12_ (cobalamin). The highly nucleophilic cobalamin (1+) ion, which is a product of the methylation of Hcy (to form methionine), commonly reacts with methyltetrahydrofolate to regenerate methylcobalamin [4]. Once the cobalt ion is oxidized by N_2_O, methylcobalamin, as a cofactor of methionine synthase in the transfer of Hcy to methionine, subsequently inhibits S-adenosylmethionine, which is essential for the methylation of myelin sheath phospholipids [5]. Thus, inactivation of vitamin B_12_ metabolism results in the demyelination of the spinal cord [6].

Few patients with cobalamin deficiency have normal serum vitamin B_12_ levels. According to the metabolic pathway described above, a normal serum level of vitamin B_12_ is not indicative of the precise or timely cellular availability of vitamin B_12_. Instead, elevated serum levels of Hcy or methylmalonic acid are better biomarkers for the diagnosis of cellular vitamin B_12_ deficiency [7]. Although the serum levels of vitamin B_12_ and folic acid returned to normal in this patient, elevated Hcy levels showed greater value as an indicator of cellular vitamin B_12_ deficiency. Thus, demyelination of the cervical spinal cord may still exist in this patient even if the serum vitamin B_12_ and folic acid levels are normal.

Moreover, the lag in the conventional MRI findings compared to clinical manifestations was similar to that seen in central pontine myelinolysis (CPM). In 1996, SCD was classified as a pure myelinolytic disease with no apparent loss of myelin or areas of partial neuropathological remyelination [8]. Hence, we surmise that the clinical-radiological dissociation observed in our case may be related to the neuropathological basis of intramedullary and interstitial edema, similar to that observed in CPM. Hyperintensity on spinal cord diffusion-weighted imaging (DWI) and a corresponding hypointensity on the apparent diffusion coefficient maps have been previously reported in patients with SCD [9, 10]. These acute demyelinating lesions manifest as restricted diffusion, indicating an energy failure, which results in cytotoxic edema.

DWI provides quantitative and qualitative functional information on the microdiffusion of water molecules at the cellular level and has been widely used for the evaluation of a variety of brain disorders, such as acute cerebral infarction [11]. Similarly, DWI is superior to T2-weighted imaging for the diagnosis of cytotoxic edema in the early stages. Hence, we hypothesize that T2-weighted imaging is not sensitive enough to reflect the early intramedullary and interstitial cytotoxic edema caused by SCD, which may be another possible reason for the clinical-imaging dissociation in the present case.

In conclusion, we recommend that N_2_O abuse should be considered in patients presenting with SCD, especially if the patient is young and otherwise healthy. The inability of serum vitamin B_12_ to reflect cellular vitamin B_12_ levels and that of T2-weighted imaging in revealing cytotoxic edema in the early stages may have contributed to the clinical-imaging dissociation. Thus, clinicians should comprehensively assess the condition of patients with N_2_O-induced SCD, avoid terminating treatment due to the resolution of clinical symptoms and serological findings, and carefully evaluate worsening imaging results for possible clinical-imaging dissociation.

## Data Availability

Data has not been made accessible in the interest of protecting the patient’s privacy.

## Abbreviations

CPM: central pontine myelinolysis
CSF: cerebrospinal fluid
DWI: diffusion-weighted imaging
Hb: hemoglobin
Hcy: homocysteine
MRI: magnetic resonance imaging
N_2_O: nitrous oxide
RBC: red blood cells
SCD: subacute combined degeneration

## References

[1] Green R, Allen LH, Bjørke-Monsen AL, Brito A, Guéant JL, Miller JW (2017). Vitamin B12 deficiency. Nat Rev Dis Primers.

[2] Patel KK, Mejia Munne JC, Gunness VRN, Hersey D, Alshafai N, Sciubba D (2018). Subacute combined degeneration of the spinal cord following nitrous oxide anesthesia: a systematic review of cases. Clin Neurol Neurosurg.

[3] Cao J, Su ZY, Xu SB, Liu CC (2018). Subacute combined degeneration: a retrospective study of 68 cases with short-term follow-up. Eur Neurol.

[4] Jordan JT, Weiser J, Van Ness PC (2014). Unrecognized cobalamin deficiency, nitrous oxide, and reversible subacute combined degeneration. Neurol Clin Pract.

[5] Hathout L, El-Saden S (2011). Nitrous oxide-induced B_12_ deficiency myelopathy: perspectives on the clinical biochemistry of vitamin B_12_. J Neurol Sci.

[6] Hunt A, Harrington D, Robinson S (2014). Vitamin B12 deficiency. BMJ..

[7] Briani C, Dalla Torre C, Citton V, Manara R, Pompanin S, Binotto G (2013). Cobalamin deficiency: clinical picture and radiological findings. Nutrients..

[8] Powers J (1996). Pathology of myelin. Mol Chem Neuropathol.

[9] Tian C (2011). Hyperintense signal on spinal cord diffusion-weighted imaging in a patient with subacute combined degeneration. Neurol India.

[10] Kim EY, Lee SY, Cha SH, Yi KS, Cho BS, Kang MH (2013). Subacute combined degeneration revealed by diffusion-weighted imaging: a case study. Clin Neuroradiol.

[11] Simonsen CZ, Madsen MH, Schmitz ML, Mikkelsen IK, Fisher M, Andersen G (2015). Sensitivity of diffusion- and perfusion-weighted imaging for diagnosing acute ischemic stroke is 97.5%. Stroke.

